# A radiosensitivity gene signature in predicting glioma prognostic via EMT pathway

**DOI:** 10.18632/oncotarget.2088

**Published:** 2014-06-10

**Authors:** Jin Meng, Ping Li, Qing Zhang, Zhangru Yang, Shen Fu

**Affiliations:** ^1^ Department of Radiation Oncology, Shanghai Jiao Tong University Affiliated Sixth People's Hospital, China; ^2^ Radiation Oncology Center, Fudan University Shanghai Cancer Center (FUSCC), Shanghai, China; ^3^ Radiation Oncology Dept, Shanghai Proton and Heavy Ion Center (SPHIC), Shanghai, China

**Keywords:** radiosensitivity, glioma, glioblastoma multiforme, gene signature, EMT

## Abstract

A 31-gene signature derived by integrating four different microarray experiments, has been found to have a potential for predicting radiosensitivity of cancer cells, but it was seldom validated in clinical cancer samples. We proposed that the gene signature may serve as a predictive biomarker for estimating the overall survival of radiation-treated patients. The significance of gene signature was tested in two previously published datasets from Gene Expression Omnibus (GEO) and The Cancer Genome Altas (TCGA), respectively. In GEO data set, patients predicted to be radiosensitive(RS) had an improved overall survival when compared with radioresistant(RR) patients in either radiotherapy(RT)-treated or non radiotherapy(RT)-treated subgroups(p<0.0001 in the RT-treated group). Multivariate Cox regression analysis showed that the gene signature is the strongest predictor(p=0.0093) in the RT-treated subgroup of patients. However, it does not remain significant (p=0.7668) in non radiotherapy-treated group when adjusting for age and Karnofsky performance score (KPS) as covariates. Similarly, in the TCGA data set, radiotherapy-treated glioblastoma multiforme(GBM) patients assigned to RS group had an improved overall survival compared with RR group(p<0.0001). Geneset enrichment analysis(GSEA) analysis revealed that enrichment of epithelial mesenchymal transition(EMT) pathway was observed with radioresistant phenotype. These results suggest that the signature is a predictive biomarker for radiation-treated glioma patients' prognostic.

## INTRODUCTION

Gliomas represent approximately 30% of primary brain tumors, and 80% of malignant tumors. Glioblastoma accounts for the majority of gliomas, while astrocytoma and glioblastoma combined account for about 75% of all gliomas.[1] Based on their histologic appearance, gliomas can be subdivided into an astrocytic (A), oligodendroglial (OD), or oligoastrocytic (OA) lineage. According to the WHO classification, they can be further subclassified into grades: I (pilocytic astrocytomas, PA), II (low grade), III (anaplastic) and IV (glioblastoma multiforme, GBM), depending on the malignant features present.[2, 3]

The response to therapy and outcome of glioma patients varies between different histological subtypes and grades.[3, 4] Most patients with WHO grade II tumours survive more than 5 years, whereas the median survival time for patients with grade III tumours is 2–3 years. Despite of the standard multimodal care for patients—surgical resection followed by adjuvant radiation therapy combined with chemotherapy, most patients with glioblastoma(WHO grade IV) succumb to the disease within one year. In the clinical setting, tumor grade is a critical factor which influences the choice of therapy modalities, particularly the use of adjuvant radiation and chemotherapy protocols. [3]

Radiation therapy(RT), as one of the major modalities of cancer therapy, plays an important role in integrated multimodality treatment for both low grade gliomas[5] and GBM[6]. Biological and technologic innovations over subsequent decades have pushed the field of radiation oncology closer toward the idealized goal of maximal local cancer control with minimal surrounding tissue toxicity.[7, 8] Emerging evidences show that new therapeutic targets have been identified to govern radioresistance in glioma.[9]

In the era of personalized medicine, prognostic and therapy-predictive molecular markers are required to guide cancer therapeutic decisions.[10, 11] One of the major issues in radiation therapy is predicting cancer radiosensitivity. At the molecular level, numerous genes have been shown to be responsive to radiation exposure. Radiosensitivity predictive assays have been developed and tested over the past few decades.[12]. As a high-throughput technology, gene signature has been used to predict radiosensitivity in many cancer types including glioblastoma, cervical, breast, colorectal, head and neck cancer cells[13-19]. One such example is the radiosensitivity index (RSI), which consists of 10 genes that associate with radiosensitivity within a collection of human cancer cell lines.[20] This signature has been clinically validated in five independent clinical data sets of different cancer type[21, 22] A similar assay identified a chemotherapy and/or radiation resistance signature using different cancer cell lines. The IFN-related DNA damage resistance signature (IRDS) analysis was evaluated retrospectively in clinical breast cancer data sets, and it successfully improved prediction of outcome after adjuvant chemotherapy and/or radiation[23].

Recently, a radiosensitivity gene signature, which includes 31 genes derived by integrating four different microarray experiments(Supplementary Table S1), has been found to have a potential for predicting radiosensitivity of cancer cells, but it was seldom validated in the clinical cancer samples. [24] In our study, we proposed that the gene signature may serve as a predictive biomarker for estimating the overall survival(OS) of radiation-treated patients. We analyzed the correlation of gene signature with overall survival time in 276 glioma patients of GSE16011 from Gene Expression Omnibus(GEO), and the prognostic value was further validated in another cohort of 463 patients with glioblastoma multiforme(GBM) from The Cancer Genome Altas (TCGA). (Supplementary Table S2,S3)

## RESULTS

### Radiosensitivity signature and Cluster analysis

**Figure 1:**
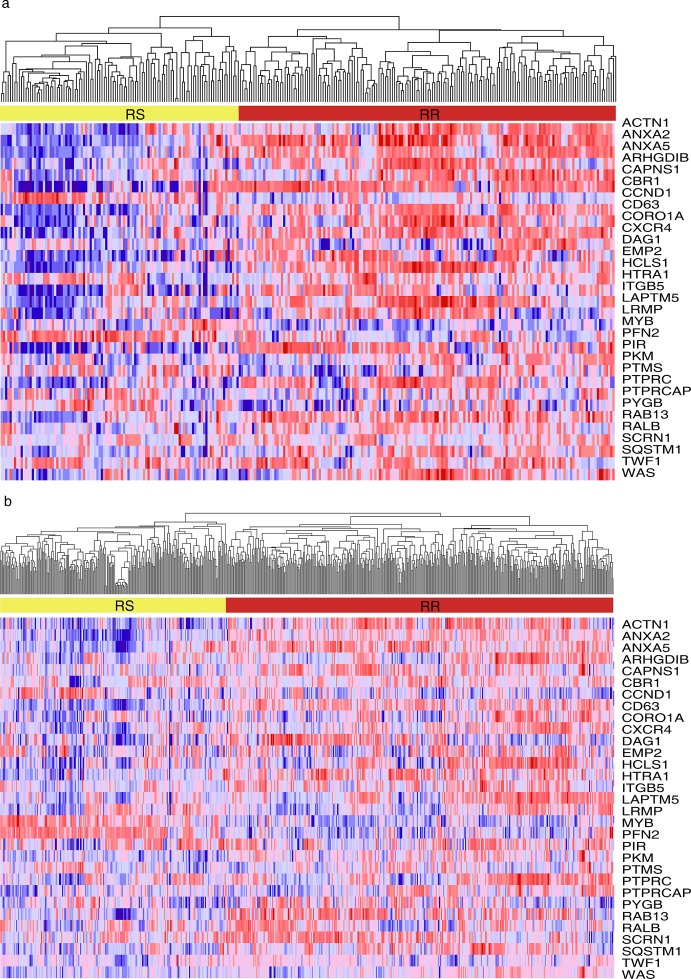
Hierarchical clustering analysis on the samples from two cohorts Hierarchical clustering was used to determine the expression pattern of 31-gene signature on the samples from GSE16011(Fig 1a) and TCGA(Fig 1b). The samples located on the left single branch of the dendrogram were subclassified as radiosensitive(RS) group, whereas the other major branch was subclassified radioresistant(RR) group according to Kim et.al's report.

The radiosensitivity molecular signature has been derived by integrating four different microarray experiments by Kim et.al[24]. Briefly, the survival fraction at 2 Gy (SF2) was used as a measure of cellular radiosensitivity. This gene set was identified with multiple microarray platforms using significant analysis of microarrays (SAM), and then gene set analysis was carried out to explore the biological processes and signaling pathways of radiosensitivity. Thus, the gene signature including 31 genes relevant to cell cycle, DNA replication, and cell junction including adherence and gap junctions was identified related to radiosensitivity. We then set out to determine the expression pattern of those 31 genes in a large panel of samples from GSE16011(Fig 1a) and TCGA(Fig1b) by using Hierarchical Clustering module in GenePattern[25]. The samples located on the left single branch of the dendrogram were subclassified as radiosensitive(RS) group, whereas the other major branch was subclassified radioresistant(RR) group according to Kim et.al's report[24].

### The association of radiosensitivity gene signature and patient's survival in GSE16011 data set

**Figure 2:**
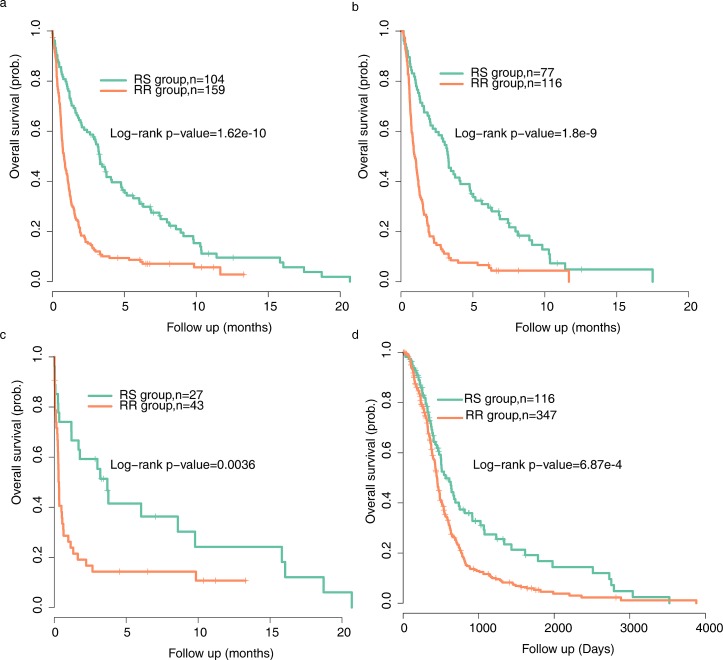
Tumor classification by radiosensitivity gene signature shows distinct prognostic outcomes Kaplan-Meier curves were used to analyze the association of the radiosensitivity gene signature with clinical outcome(overall survival) of (a) all glioma patients from GSE16011(n=263) (b) patients treated with radiation therapy from GSE 16011(n=193) (c) patients treated with no radiation therapy from GSE 16011(n=70) (d) GBM patients from TCGA(n=463).

The association between radiophenotype(as predicted by the radiosensitivity gene signature) and clinical outcome was examined for all the patients with glioma in GSE 16011. Patients predicted to be radiosensitive(RS) are expected to have better survival outcomes compared with patients predicted to be radioresistant(RR). Using the radiosensitivity gene signature, 263 patients were divided into RS(n=104) and RR (n=159) groups. Patients in the RR group had significantly shorter overall survival than those in the RS group (log-rank test P<0.0001)(Figure 2a).

### The radiosensitivity model predicts clinical outcome only in RT-treated patients

In order to further determine whether the signature is RT-specific, we conducted a subgroup analysis in the dataset. Kaplan–Meier curves were performed for patients treated with and without radiotherapy(RT) separately. RS patients had a superior overall survival compared with RR patients either in radiotherapy-treated subset or in the patients subset that did not receive RT (Figure 2b,<0.0001 in the RT-treated goup and Figure 2c,p=0.0036 in non RT-treated group).

Nevertheless, in the multivariate Cox regression analysis to assess for independent predictors of the relation between the gene signature and clinicopathologic features, we found that the gene signature is the strongest predictor(p=0.0093) in the subgroup of patients with radiotherapy, whereas it does not remain significant (p=0.202) in the non RT group when taking age and Karnofsky performance score (KPS) into account.(Table 3) Taken together, the radiosensitivity gene signature is mainly predictive in patients treated with radiation therapy.

**Table 1:** Patient demographics and clinical characteristics of GSE16011 cohort(n=276)

Characteristic	No.(available)	%
Gender		
Male	184	66.7
Female	92	33.3
Age at diagnosis,years		
Mean	50.3	
Std. Deviation	14.71	
KPS		
Mean	80.68	
Std. Deviation	19.55	
Survival years		
Mean	2.73	
Std. Deviation	3.52	
WHO grade and histology		
I(PA)	8	2.9
II	24	8.7
A	13	4.7
OA	3	1.1
OD	8	2.9
III	85	30.8
A	16	5.8
OA	25	9.1
OD	44	15.9
IV(GBM)	159	57.6
Treatment		
Radiotherapy		
No	78	28.3
Yes	198	71.7
Additional chemotherapy		
No	173	62.7
Yes	27	9.8
Type of surgery		
Complete resection	86	31.2
Partial resection	39	14.1
Biopsy	146	52.9

**Table 2:** Patient demographics and clinical characteristics of TCGA(The Cancer Genome Atlas) cohort(n=463)

Characteristic	No. (available)	%
Gender		
Male	288	62.2
Female	175	37.7
Age at diagnosis,years		
Mean	56.35	
Std. Deviation	13.99	
KPS		
Mean	81.9	
Std. Deviation	14.09	
Molecular subtype	435	
proneural	112	24.2
neural	64	13.8
classical	126	27.2
mesenchymal	131	28.3
Additional chemotherapy	408	
No	226	48.9
Yes	182	39.3
Additional surgery procedure	217	
No	97	21
Yes	120	25.9

**Table 3:** Multivariate Cox regression analysis of RT-treated patients in GSE16011 patients. HR = hazard ratio; CI = confidence interval

Covariate	P	HR	95% CI
All the patients with glioma(n=276)			
Gene signature	0.012	2.217	1.193 to 4.121
Age at diagnosis	0.021	1.029	1.005 to 1.053
KPS	0.274	0.988	0.967 to 1.010
WHO grade	0.486	1.416	0.534 to 3.753
Reviewed histologic diagnosis	0.514	1.205	0.690 to 2.107
Chemotherapy	0.794	0.932	0.549 to 1.580
Type of surgery	0.063	1.345	0.986 to 1.833
IDH1_ mutation	0.111	0.557	0.272 to 1.141
co 1p/19q mutation	0.277	1.455	0.743 to 2.849
RT-treated patients			
Gene signature	0.009	2.325	1.236 to 4.374
Age at diagnosis	0.022	1.029	1.004 to 1.054
KPS	0.295	0.988	0.966 to 1.010
WHO grade	0.315	1.702	0.606 to 4.780
Reviewed histologic diagnosis	0.541	1.205	0.665 to 2.183
Chemotherapy	0.453	0.804	0.456 to 1.417
Type of surgery	0.049	1.383	1.003 to 1.906
IDH1_ mutation	0.091	0.525	0.250 to 1.103
co 1p/19q mutation	0.675	1.161	0.579 to 2.329
Non RT-treated patients			
Gene signature	0.202	1.510	0.805 to 2.831
Age at diagnosis	0.002	1.035	1.013 to 1.058
KPS	0.002	0.974	0.959 to 0.990

### The radiosensitivity model predicts clinical outcome in TCGA cohort

Given the clinical importance of correct assignment, we tended to validate our results using more clinically homogenous dataset with different chip platforms. The TCGA cohort consists of 463 patients with histologically confirmed glioblastoma multiforme (GBM, WHO grade IV). All the patients had received radiation therapy. Similar to the previous findings, patients predicted to be RR had shorter survival time than patients predicted to be RS(Figure 2d). In consistence with the results described above, patient's survival in the RS group was better than that in the RR group throughout the follow-up. The effect of gene signature group, clinical factors as age, KPS, molecular subtype and treatment procedures on GBM patient survival time was further evaluated by multivariate Cox proportional hazard model. The results showed that gene signature might be an independent predictor of patient survival (Table 4). Further Cox regression analysis on each subtype of samples were carried out and the results were shown as Supplementary Figure S1.

**Table 4:** Multivariate Cox regression analysis of RT-treated patients in TCGA patients. HR = hazard ratio; CI = confidence interval

Covariate	P	HR	95% CI
Gene signature	0.033	1.897	1.055 to 3.413
Age at diagnosis	0.003	1.023	1.008 to 1.039
Karnofsky_performance_score	0.336	0.993	0.979 to 1.007
Additional chemotherapy	0.019	0.633	0.432 to 0.928
Additional surgery procedure	0.857	0.965	0.656 to 1.421
Gene Expression Subtype	0.417	0.920	0.751 to 1.126

### Identification of EMT gene sets involved in gene signature

**Figure 3:**
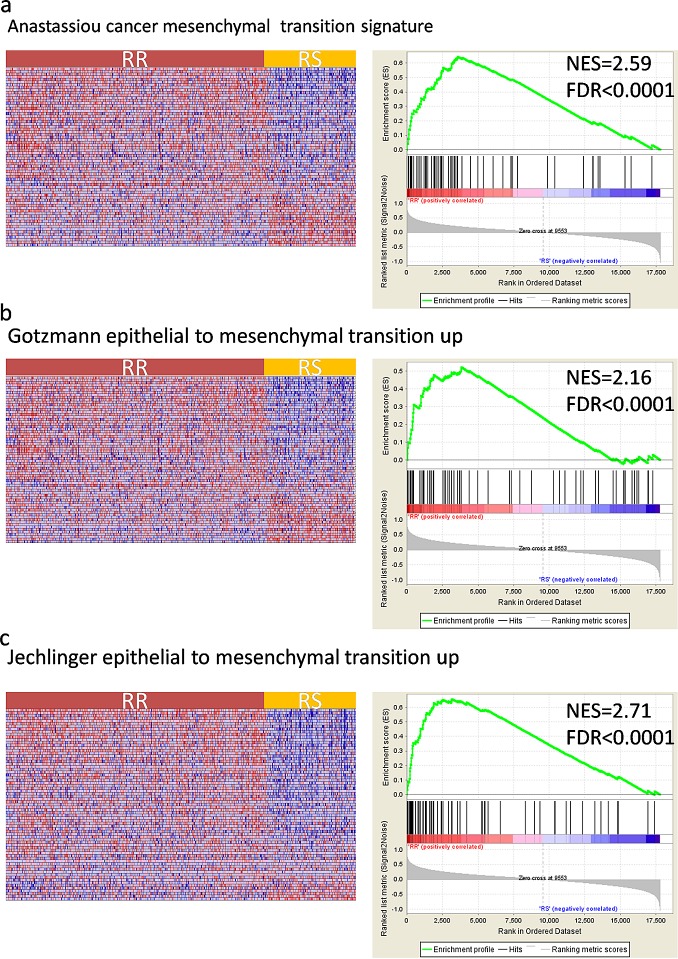
Gene Set Enrichment Analysis(GSEA) shows enrichment of EMT(epithelial mesenchymal transition) related genes among radioresistant(RR) patient classified by the gene signature GSEA validated enhanced activity of (a) Anastassiou cancer mesenchymal transition signature (b) Gotzmann epithelial to mesenchymal transition up (c) Jechlinger epithelial to mesenchymal transition up. The horizontal bar in graded color from red to blue represents the rank-ordered, non-redundant list of genes. The vertical black lines represent the projection of individual genes onto the ranked gene list. Genes on the left side (red) correlated most strongly with increased EMT related gene expression. NES, normalized enrichment score; FDR, false discovery rate.

Epithelial mesenchymal transition(EMT) is known as a facilitator of cellular dissociation and migration, which plays a critical role in caner metastasis. Recently, EMT was reported to be related to radioresistence in many cancers[26, 27] and specifically targeting EMT may provide a new targeted approach for improving the therapeutic effectiveness of radiation in cancers[28]. To understand whether the RR samples were enriched with EMT related pathway, we performed Gene Set Enrichment Analysis(GSEA) in TCGA cohort. GSEA is a computational method that assesses coordinate expression changes at a pathway level. To assess the direct transcriptional targets, EMT related gene sets were obtained using the GSEA tool from MIT (www.broad.mit.edu/gsea). As seen in Fig. 3, several gene sets of epithelial mesenchymal transition(EMT) were associated with radioresistant phenotypes. Collectively, these data suggest that radioresistant phenotype was enriched for genes of EMT, whereas radiosensitive phenotype correlated strongly with decrease of genes of EMT.

## DISCUSSION

In the “omics” era, the generation of high-throughput datasets has been widely used to better define treatment and disease outcome. As a genetic disease, cancer is composed of multiple molecular alterations. Aiming at improving cancer care, it is important to examine and try to understand these genetic alterations. Over the last 15 years, gene signatures with specificity in terms of diagnosis, prognosis or prediction of therapy response have been developed and validated by different techniques and teams.[29] For instance, one of the first groups reported a prognostic gene signature in breast cancer.[30] The 70-gene signature provided prognostic and predictive information, and it further triggered development of commercial test(Mammoprint). The prognostic value of Mammoprint has been validated in a series of patients including response prediction for chemotherapy in breast cancer.[31-34] Gene expression profiles have also been identified for classification and prognosis in gliomas.[35-37] However, few radiation-specific biomarkers have become routine due to the lack of validation in clinical radiation oncology practice. Kim et al. has integrated four different microarray and identified genes as candidates for radiosensitivity biomarkers. The gene signature involves 31 genes related to cell cycle, cell junctions, and cell adhesion. In this paper, we propose this discovery platform as a rational strategy to the identification of novel radiation-specific biomarker for gliomas. We test the gene signature in two independent data sets of glioma patients, revealing that the gene signature were significantly associated with survival endpoints. The combined clinical and laboratory data strongly indicate that the 31-gene signature is principally a therapy-predictive marker for glioma patients.

We first examined whether there was any association between radiosensitivity gene signature and clinical outcome in glioma patients from GSE16011 cohort. Patients were then subdivided into RT-treated and non RT-treated groups. By applying the 31 gene signature to the RT-treated patients, we observed a clear separation in the survival curves between patients predicted to be radiosensitive and radioresistant. Patients predicted to be RS in their tumor specimens tended to have prolonged overall survival, whereas patients predicted to be RR tended to have shortened survival. Interestingly, there was no difference in outcome between predicted RS and RR patients that did not receive RT after multivariable Cox regression analysis suggesting that the gene signature is RT-specific. Due to the lack of treatment homogeneity in GSE16011 data set, we further validate the gene signature in another data set obtained from TCGA. For the TCGA data set, all the patients have been diagnosed glioblastoma multiforme histologically, and have been treated with radiotherapy. The association of radiosensitivity gene signature and clinical outcome was also analyzed, and the results were similar to those observed in glioma patients above. Given the clinical importance of correct assignment, any molecular biomarkers have to be confirmed not only with different technical platforms but also with external patient populations. As a confirmatory step, our data show that the radiotherapy specific molecular subgroups correlate with patient survival.

Further analysis revealed that the prognostic value of the radiosensitivity signature was independent of age and KPS, the strongest survival predictors in patients suffering from glioma. In general, younger age and higher KPS show better survival, whereas older age and lower KPS is correlated with worse survival in a treatment independent manner. [38] In accordance with other studies, age was a significant prognostic factor in our study when assessed in the multivariable Cox regression analysis. In the stratification analysis, when age and KPS were analyzed simultaneously by multivariate analysis, significant associations of gene signature and clinical outcome were no longer present for non-RT treated subgroup in GSE data set. Traditionally, prognosis of a patient with GBM is associated with WHO grade and histology. [3] Other generally accepted predictors of survival are the extent of tumor resection, additional chemotherapy, additional surgery locoregional procedure.[39]

Today's advancements in genetics technology have surfaced more molecular associations with outcomes for these patients, such as IDH1 mutation, codeletion of chromosome 1p/19q, MGMT promoter methylation status.[5, 39, 40] Patients with codeletion of 1p and 19q have an increased response to treatment and favorable outcomes. [41]Isocitrate dehydrogenase 1 (IDH1) mutations are also indicated in increased survival in GBM patients. Patients with MGMT promoter methylation and IDH1 mutation are associated with increased progression-free and overall survival.[42, 43] Here, by performing multivariable Cox regression analysis, we showed that the radiosensitivity gene signature is independent of these possible prognostic predictors(when available) among patients of glioma treated with radiotherapy.

In addition, using GSEA analysis, we find strong evidence for the upregulation of EMT gene sets associated with radioresistant phenotype. As indicted by Kim et al, among the 31 genes, ACTN1, CCND1, HCLS1, ITGB5, PFN2, PTPRC, RAB13, and WAS, totally 8 genes, are adhesion-related molecules, which are enriched in the integrin and EMT signaling pathway. [24] Integrins are molecules that directly bind to the extracellular matrix(ECM) and regulate diverse functions in tumour cells, including migration, invasion, proliferation and survival. Moreover, Integrins are instrumental in the activation and modulation of TGFβ signaling, which is a well-characterized inducer of epithelial–mesenchymal transition(EMT). [44] By performing GSEA Analysis, we successfully validated that several gene sets of epithelial mesenchymal transition(EMT) were associated with radioresistant phenotypes. Based on these results, we might speculate that the gene signature might be involved in the epithelial–mesenchymal transition process. Epithelial–mesenchymal transition (EMT), characterized by a cellular process during which epithelial cells lose their polarized organization and cell–cell junctions, plays a critical role not only in tumor metastasis but also in tumor recurrence. [45] Emerging evidence suggests that EMT plays a crucial role in cancer radiation resistance. [46, 47] However, the role of EMT in glioma radioresistance remains elusive. In clinical setting, resistance to radiotherapy is a major obstacle to the effective treatment of glioma, our findings may bring a new broad of perspective for new therapeutic targets.

In summary, our study demonstrates the prognostic values of radiosensitivity gene signature identified by Kim et al. Our data suggest that the signature is a predictive biomarker of radiotherapy treatment benefit for glioma patients. Epithelial mesenchymal transition(EMT) pathway might be associated with radioresistant phenotypes classified by the gene signature. Future prospective studies will be needed to fully refine the integrated prognostic algorithm in clinical radiation oncology.

## METHODS

### GEO glioma patients gene expression data

The largest data set (GSE16011) of glioma patients from the publicly available GEO databases was used as one of the validation set. Gene expression data and corresponding clinical data used in this study were obtained from GEO databases and related article [48]. The study subjects in this cohort were collected from the Erasmus University Medical Center tumor archive (n = 276) from patients between 1989 and 2005, which has been previously described. [48] The cohort consists of 276 patients with histologically confirmed gliomas of different grades: 8 astrocytomas grade I (PA), 24 grade II(13 astrocytomas, 3 OA and 8 OD), 85 grade III(16 astrocytomas, 25 OA and 44 OD), 159 astrocytomas grade 4 (GBM) Male-to-female ratio was 2:1, median age at diagnosis was 50.3 years (range, 11.7–81.2), and mean KPS was 80.7. Extended demographics for these patients are shown in Table 1. Thirteen patients with no survival information were excluded in the analysis(n=263).

### TCGA glioma patients gene expression data

For the TCGA cohort, the gene expression profiles were studied in 463 GBM tumors from patients who were chosen from the updated TCGA database(All the data were available without limitations as assessed on Nov.21,2013). [49] Only patients having received radiation therapy and intact OS information were included in the study. A total of 463 GBM samples of following molecular subtype were included in this study: 128 classical, 133 mesenchymal, 65 neural, 113 proneural(others NA). Male-to-female ratio was 1.6:1, median age at diagnosis was 56 years (range, 10–86) and mean KPS was 78.5. Detailed patient characteristics are listed in Table 2.

### Microarray data processing

RNA preparation procedure has been previously described. [48, 49] Raw gene expression data for both datasets are publicly available in GEO (http://www.ncbi.nlm.nih.gov/geo/) and TCGA(http://cancergenome.nih.gov/) Affymetrix HU133 Plus 2.0 arrays was used for GEO cohort, whereas The Cancer Genome Atlas (TCGA) data sets used HU133A microarrays.

### Statistical analysis

The analysis was conducted for all patients in each dataset. Patients were divided into RS and RR groups, as described in the previous study. [24] This RS/RR variable was compared with OS of each dataset. The Kaplan-Meier method was used to estimate survival time for the RS/RR groups, along with the two-sided log-rank test to determine the difference between the two groups. Cox proportional hazard models was fit to obtain HRs. Furthermore, we used Cox multivariate analysis to test whether the RS/RR group was an independent predictor for survival time. All the data was analysed by SPSS (version 16.0), The significance level was defined as 0.05.

### Gene set enrichment analysis (GSEA)

GSEA was performed by the JAVA program (http://www.broadinstitute.org/gsea) using MSigDB C2 curated gene set collection. Gene sets with a false discovery rate(FDR) value <0.05 after performing 1,000 permutations were considered to be significantly enriched. [50]

### Competing interests

The authors declare that they have no competing interests in this study.

## SUPPLEMENTARY MATERIALS AND FIGURES




