# Supplementary material for: A radiosensitivity gene signature in predicting glioma prognostic via EMT pathway
Source: Oncotarget. 2014 Jun 10;5(13):4683–93. doi: 10.18632/oncotarget.2088 (PMC4148091; doi:10.18632/oncotarget.2088)
Supplement: Supplementary file 2 [file oncotarget-05-4683-s002.pdf]

A radiosensitivity gene signature in predicting glioma prognostic via EMT pathway

Supplementary Material

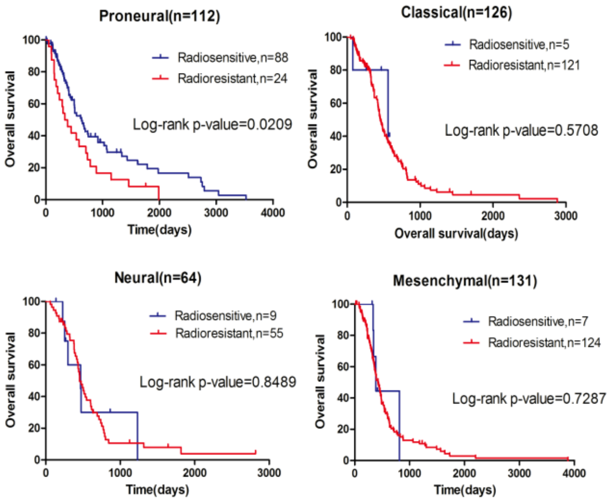

Suppl figure-1: Cox regression analysis on each subtype of samples in TCGA
